# Does Government subsidy for costs of medical and pharmaceutical services result in higher service utilization by older widowed women in Australia?

**DOI:** 10.1186/1472-6963-12-179

**Published:** 2012-06-27

**Authors:** Leigh R Tooth, Richard Hockey, Susan Treloar, Christine McClintock, Annette Dobson

**Affiliations:** 1 School of Population Health, The University of Queensland, Brisbane, Australia; 2 Centre for Military and Veterans’ Health, The University of Queensland, Brisbane, Australia

## Abstract

**Background:**

In Australia, Medicare, the national health insurance system which includes the Medical Benefits Scheme (MBS) and Pharmaceutical Benefits Scheme (PBS), provides partial coverage for most medical services and pharmaceuticals. For war widows, the Department of Veterans’ Affairs (DVA) covers almost the entire cost of their health care. The objective of this study was to test whether war widows have higher usage of medical services and pharmaceuticals.

**Methods:**

Data were from 730 women aged 70–84 years (mostly World War II widows) participating in the Australian Longitudinal Study on Women’s Health who consented to data linkage to Medicare Australia. The main outcome measures were PBS costs, claims, co-payments and scripts presented, and MBS total costs, claims and gap payments for medical services in 2005.

**Results:**

There was no difference between the war widows and similarly aged widows in the Australian population without DVA support on use of medical services. While war widows had more pharmaceutical prescriptions filled they generated equivalent total costs, number of claims and co-payments for pharmaceuticals than widows without DVA support.

**Conclusions:**

Older war widows are not using more medical services and pharmaceuticals than other older Australian women despite having financial incentives to do so.

## Background

In Australia, an in-situ experiment in older women’s use of medical services and pharmaceuticals can inform health service policy on the effectiveness of alternative models of health funding. Different models of health service funding and charging are applied for medical services and pharmaceuticals under Australia’s universal health care system (‘Medicare’), and the special health care coverage provided to Australia’s war veterans and their widows.

Medicare ensures eligible Australian residents have access to free treatment as a public patient in public hospitals, free or low cost out-of-hospital medical and optometric care and subsidized access to pharmaceuticals. Residents may choose to pay for private insurance for hospital and other health services not covered by Medicare (e.g., allied health). For each out-of-hospital visit to a general (family) practitioner (GP) (equivalent to U.S. primary care physician) or medical specialist, Medicare pays 85 to 100 % of the Medicare Benefits Schedule (MBS) fee, depending on the consultation type (MBS fees are set annually by Government), potentially leaving a ‘gap’ payable by the patient. Many practitioners charge more than the schedule fee; the difference between the Medicare benefit and the practitioner’s actual charge is the patient’s eventual out-of-pocket cost. The basic architecture of the Medicare system is shown in Table 1 and while a minority of Australian residents incurs some cost for health care, there are levels of protection safeguarding individuals with excessive health care needs against paying too much.

**Table 1:** Arrangements for out-of-hospital medical (MBS) and pharmaceutical (PBS/RPBS) services in Australia for individuals with and without DVA Gold Cards

**All Australian residents**	**Australian residents with DVA Gold Card**
**Medicare**	**Medicare**
· Medicare pays 85-100% of scheduled fee for GP/specialist visit up to threshold (see below)	· Always bulk-billed i.e., no gap or out-of-pocket costs
· Person pays gap of up to 15% plus out-of-pocket costs (difference between scheduled fee and GPs/specialist’s charge)	· DVA pays GPs higher fees according to its own schedule
· GPs/Specialists may accept Medicare payment (bulk billing). This is most common for concession card holders and in areas of lower SES	· Covers a comprehensive range of Allied and Dental Health services
	· Provides for rehabilitation appliances
	· Funds in home nursing and domestic support services
· **Medicare Safety Net threshold (2012 Figures)**:	
· All Medicare card holders (Australian residents)	
· Once $413.50 for gap payments has been met annually, Medicare will pay 100% of scheduled fee	
· Once $1198.00 for out-of-pocket costs for extended Medicare Safety Net services has been met annually, Medicare will pay 80% of out-of- pocket costs	
· Concession card holders – Once minimum $598.80 for out-of-pocket costs for extended Medicare Safety Net services has been met annually, Medicare will pay 80% of out-of-pocket costs	
**PBS Co-payment**	**PBS/RPBS Co-payment**
· General patient - $34.40 per scripts up to threshold	· $5.80 per script up to threshold of $348.00
· Concession card holder - $5.80 per script up to threshold	
**PBS Safety Net threshold (2012 Figures)**:	**PBS Safety Net threshold**:
· All Medicare card holders (Australian residents) - Once $1363.30 has been spent annually on pharmaceuticals listed on the PBS, further pharmaceuticals are maximum $5.80	· Once minimum $ 348.00 has been spent annually on pharmaceuticals listed on the PBS/RPBS, further pharmaceuticals are free
· Concession card holders – Once $348.00 has been spent annually on pharmaceuticals listed on the PBS, further pharmaceuticals are free	

PBS – Pharmaceutical Benefits Scheme; RPBS – Repatriation Pharmaceutical Benefits Scheme; SES - Socio-economic status.

The Australian Government also subsidizes the costs of many prescription medicines for residents through the Pharmaceutical Benefits Scheme (PBS), also illustrated in Table 1. Individuals pay a maximum co-payment per script for prescription medications listed on the PBS, regardless of their actual cost. The level of co-payment is lower for concession card holders such as pensioners. There is also a PBS Safety Net to protect individuals who require numerous medicines.

Separately, the Australian Government funds the Department of Veterans’ Affairs (DVA), to provide benefits and services to eligible veterans and their dependants for injury, disease or death related to Australian Defence Force service (similar to the U.S. Civilian Health and Medical Program of the Department of Veterans’ Affairs (CHAMPVA)). War widows are entitled to a war widow’s pension and gold repatriation health card (Gold Card) from the DVA if their husband’s death was due to war service or other eligible defence service. With a Gold Card, war widows are always bulk billed (meaning that they pay nothing personally) and the benefit paid is higher. War widows are also entitled to receive from DVA more health services than Medicare covers for other Australians, for example all allied health services and private hospital costs. War widows with DVA Gold Cards are entitled to all medicines listed on the PBS and the Repatriation Pharmaceutical Benefits Scheme (RPBS) at the concessional co-payment rate. The RPBS includes some additional pharmaceuticals specifically for the veteran population. Thus, war widows are a unique group of Australian women. Regardless of their own health needs, once their spouse dies they receive their own Gold Card if they are eligible, which entitles them to a wider range of free medical and other health care (including treatment in private and public hospitals) and a larger range of pharmaceuticals at the concessional rate than other similarly aged Australian women.

Research has shown that abolishing or reducing the costs of health care leads to higher usage of health services [1]. As financial disincentives for using health care services are largely abolished for war widows, it might be expected that they would also make higher use of these services, particularly as widows often have poorer health, higher mortality and higher use of health services compared to partnered women [2,3]. Little research has examined this issue.

For this paper, we hypothesized that war widows with Gold Cards would use more medical services and pharmaceuticals than widows without a connection to DVA. To test this, we analysed data from a large community-based cohort study of older Australian women.

## Methods

### Sample and data

The data were from the Australian Longitudinal Study on Women’s Health (ALSWH) which involves over 40,000 women randomly selected in 1996 from the Medicare Australia database which includes most of the Australian population. Women from rural/remote areas were sampled at twice the rate of women in urban areas to ensure adequate ongoing representation as the study progressed. Three age cohorts were selected, women born between 1973–78, 1946–51 and 1921–26 and these women complete omnibus-style mailed surveys on their health and social circumstances every three years (see http://www.alswh.org.au/surveys.html). The National Death Index is checked annually to ascertain deaths.

The ALSWH was chosen because it is the largest longitudinal study of women in Australia and is largely representative of the general population of Australian women [4-6]. The ALSWH has ethical clearance from the University of Newcastle, the University of Queensland, the Australian Department of Health and Ageing and the Department of Veterans Affairs (DVA). This allows linkage of ALSWH and Medicare Australia data for women who consent. For this paper, data from the fourth ALSWH survey (in 2005) of women born between 1921–1926, then aged 79–84 years were used. In this survey, each woman was asked whether she had a DVA Gold Card and, if married, whether her spouse had a DVA Gold Card. She was also asked about the sources of income for her and her husband with options that included all relevant DVA pensions. The women’s marital status (married, defacto, widowed, single, divorced, separated) is asked at every ALSWH survey. Widows are asked the date they were widowed. Only Australian-born women who were married at Survey 1 and widowed by Survey 4 were included.

The women were categorized as war widows with DVA Gold Cards or widows with no connection to DVA. The women were further categorized by duration of widowhood as this is associated with health and lifestyle changes, for example poorer mental and physical health, higher mortality and difficulty managing on income [7,8]. Given the 3-yearly cycle of ALSWH surveys, duration of widowhood was defined as being recent (≤3 years) or longer (>3 years).

In the ALSWH surveys, consent is sought from all participants for Medicare Australia (which also processes Gold Card payments on behalf of DVA) to release linkable claim details to the ALSWH research team. All claims for services (including MBS/PBS/RPBS) that were processed by Medicare Australia for consenting women for 2005 (same year as fourth ALSWH survey) were extracted. The outcomes used in this study were: total cost of medical services (including gap payment made by women); total number of claims for MBS subsidized medical services; total gap payments; number of GP visits; total costs of pharmaceuticals (PBS/RPBS); number of pharmaceutical claims; number of pharmaceutical scripts presented; and total co-payments for pharmaceuticals. Table 2 presents further explanations about the outcomes.

**Table 2:** Definitions and further explanations of the medical services and pharmaceutical outcomes

**Outcome variable**	**Interpretation of variable and scores**
**Medical Services**	
Total costs of medical services	Total cost of medical services for 2005 calendar year (includes Medical Benefit Scheme (MBS) gap payment) ($AU)
Number of MBS claims	Total number of claims to MBS for medical services for 2005 calendar year (additional bulk billing payments for general medical services were excluded from claims data to prevent double counting)
MBS gap payment by the women	Total amount paid by women for medical services for 2005 calendar year (AU$). Due to skewness this variable is dichotomised into whether a gap was paid (yes, no).
Number of General Practitioner (GP) visits	Total number of un-referred visits to GPs for 2005 calendar year
**Pharmaceuticals**	
Total costs of pharmaceuticals (PBS/RPBS)	Total cost of pharmaceuticals for 2005 calendar year (includes women’s contribution) ($AU)
Number of PBS(RPBS) claims	Total number of scripts dispensed (including each repeat dispensed on one script) in 2005 calendar year
Number of PBS(RPBS) scripts presented	Total number of scripts presented (irrespective of how many repeats per script) in 2005 calendar year
Women’s contribution to cost of pharmaceuticals	Total amount paid by women in 2005 calendar year ($AU).

Comparison of ALSWH data with linked Medicare data enabled women’s descriptions of their connection to DVA to be cross-checked with the concession status of their MBS/PBS(RPBS) claims.

### Analysis

War widows with DVA Gold Cards (≤3 years or >3 years widowed) and widows with no connection to DVA (≤3 years or >3 years widowed) were compared on the MBS/PBS(RPBS) outcomes, after controlling for the potential confounders of age, self-reported ability to manage on their income and self-rated health. For the continuous MBS/PBS(RPBS) outcomes, multiple regression analyses and selected pair wise comparisons were conducted. The mean number (95 % confidence intervals (CIs)) of MBS claims, GP visits, PBS/RPBS claims and scripts are presented, and the mean total (95 % CIs) costs for medical services, pharmaceuticals and the women’s co-contribution to pharmaceuticals are reported. For the MBS gap payment, this outcome was dichotomized into whether a gap payment was made or not as the data were skewed. Logistic regression was used for this analysis and odds ratios (95 % CIs) are reported. Analyses were conducted using SAS (version 9.2).

## Results

The fourth ALSWH survey was completed by 7158 women. Of these, 1534 (21.4 %) were married at Survey 1 and widowed by Survey 4. Of these, 999 (65 %) consented to Medicare linkage. The final sample was the 730 women for whom their relationship with DVA could be correctly ascertained and who were Australian-born. Of these 730 women, 407 (56 %) had no connection to DVA and 323 (44 %) were DVA Gold Card holders. Table 3 shows selected demographic characteristics of the 730 women. The groups differed statistically on ability to manage on available income. The strongest contributor to this result was that longer-term widows without a connection to DVA were more likely to report having difficulty managing on their income. The groups did not differ statistically on age or self-rated health.

**Table 3:** Demographic characteristics of Australian Longitudinal Study on Women’s Health participants: Grouped by relationship to the Department of Veterans’ Affairs (DVA) and recency of widowhood

**Demographic characteristic**	**Gold Card holder, widowed ≤ 3 years n = 114**	**Gold Card holder, widowed > 3 years n = 209**	**Not DVA, widowed ≤ 3 years n = 136**	**Not DVA, widowed > 3 years n = 271**	**p-value**
Age (mean, SD)	81.1 (1.4)	81.3 (1.4)	80.9 (1.4)	81.1 (1.4)	0.14
Self-rated heath (%)					
Excellent	0.9 %	4.9 %	4.4 %	6.6 %	0.12
Very good	15.0 %	21.8 %	22.2 %	24.0 %	
Good	53.1 %	39.8 %	45.2 %	43.5 %	
Fair/Poor	31.0 %	33.5 %	28.1 %	25.8 %	
Ability to manage on income (%)					
Easy	38.6 %	43.7 %	32.1 %	30.6 %	
Not too bad	44.7 %	46.1 %	53.7 %	45.1 %	
Difficult sometimes	13.2 %	9.7 %	11.2 %	17.2 %	0.001
Difficult always/ impossible	3.5 %	0.5 %	3.0 %	7.1 %	

### Medical services: Total costs, number of claims, visits to GPs and gap payment

No large or statistically significant differences between the groups were found for total costs of medical services (p = 0.64), number of MBS claims (p = 0.08) and visits to GPs (p = 0.21) (Table 4). Regarding gap payments, 5 to 10 % of the war widows in this study still chose to pay a gap despite theoretically not needing to. However overall, these Gold Card holders were much less likely to incur gap payments: the adjusted odds ratios ranged from 46 (95 % CIs 21, 102) for recent widows to 76 (95 % CIs 37, 156) for longer-term widows (Table 5).

**Table 4:** Medical Benefit Scheme (MBS) and Pharmaceutical Benefits Scheme (PBS/RPBS) items to Australian Longitudinal Study on Women’s Health participants by Department of Veterans’ Affairs (DVA) and partnership status: Mean (95 % CIs) total MBS costs, MBS claims, PBS/RPBS costs, PBS/RPBS claims and PBS/RPBS scripts presented

	**Gold Card holder, widowed ≤ 3 years n = 114**	**Gold Card holder, widowed > 3 years n = 209**	**Not DVA, widowed ≤ 3 years n = 136**	**Not DVA, widowed > 3 years n = 271**	**p-value**
**MBS**					
Total costs ($AU: mean 95 % CIs)	1213 (968,1521)	1070 (904,1268)	1265 (1027,1556)	1166 (1006,1352)	0.64
Claims (number: mean 95 % CIs)	25.4 (21.7,29.6)	21.3 (19.0,24.0)	26.9 (23.3,31.0)	24.0 (21.7,26.6)	0.08
GP visits (number: mean 95 % CIs)	10.6 (9.4,11.9)	9.7 (8.9,10.7)	9.6 (8.6,10.7)	9.1 (8.4,9.8)	0.21
Gap payment (% yes)	9.8 %	4.9 %	81.3 %	76.6 %	<0.0001
**PBS(RPBS)**					
Total costs ($AU: mean 95 % CIs)	998 (816, 1220)	1024 (879, 1192)	1020 (845, 1231)	868 (761, 989)	0.33
Claims including repeats (number: mean 95 % CIs)	38.8 (33.5,45.0)	38.7 (34.6,43.3)	38.1 (33.2,43.8)	32.9 (29.9,36.3)	0.10
Scripts presented (number: mean 95 % CIs)	15.3 (13.4,17.4)	15.2 (13.7,16.7)	14.5 (12.8,16.4)	12.2 (11.2,13.3)	0.001
Co-contribution ($AU: mean 95 % CIs)	149 (129, 172)	151 (136, 169)	168 (146, 192)	152 (138, 167)	0.54

All analyses adjusted for age, self-rated health and ability to manage on income.

**Table 5:** Odds ratios for no MBS gap payment in 2005 calendar year for the groups defined by relationship with Department of Veterans’ Affairs and recency of widowhood

**Comparison Groups**	**Odds ratio**	**95 % CIs**	**p-value**
Gold Card holder, widowed > 3 years versus Gold Card holder, widowed ≤ 3 years	2.09	0.85, 5.10	0.11
Not DVA, widowed > 3 years versus not DVA, widowed ≤ 3 years	1.28	0.75, 2.20	0.36
Gold Card holder, widowed ≤ 3 years versus not DVA, widowed ≤ 3 years	46.5	21.3, 101.9	<0.0001
Gold Card holder, widowed > 3 years versus not DVA, widowed > 3 years	75.5	36.6, 155.9	<0.0001

Adjusted for age, self-rated health and ability to manage on income

### Pharmaceuticals: Total costs, number of claims, number of scripts and amount of co-contribution

No large or statistically significant differences were found between groups for total costs of pharmaceuticals (p = 0.33), total number of claims for pharmaceuticals (p = 0.10) and amount of the women’s co-contribution to the costs of their pharmaceuticals (p = 0.54) (Table 4). A significant difference was found between the groups for presentation of PBS scripts. Subsequent pair-wise analysis revealed the contributing factor to this result was that for longer-term widows, those with no connection to DVA presented fewer PBS(RPBS) scripts than women who were DVA Gold Card holders (pair wise p = 0.001) (Table 4).

## Discussion

We hypothesised that war widows with Gold Cards would use more medical services and pharmaceuticals than women without a connection to DVA, thereby costing the Australian Government more. This hypothesis was based on the premise that the removal of financial disincentives would lead to higher use of services. This hypothesis was not substantiated. DVA support reduced the out-of-pocket costs for medical services. However despite having access to gap free treatment, Gold Card holders neither incurred higher total costs, nor made more claims for medical services or visits to GPs. Indeed, between 5–10 % of war widows in this study did pay a gap. These findings suggest they are not making increased use of almost free medical treatment. In an analysis of GP visits by people aged over 70 years before and after free GP services were introduced, Layte et al [9] found no significant increases in number of GP visits. He suggested that the impact of other factors such as personal mobility, transport and information may be more important than costs in determining frequency of GP visits. In an analysis of factors determining use of GPs in Australia, Zhang and colleagues further identified issues such as social support, perceptions about the value of good health and attitudes about health care as being important [10].

Having a Gold Card did appear to influence use of pharmaceuticals by war widows in a limited way. These Gold Card holders, in particular longer-term widows, presented more PBS(RPBS) scripts than women who were not Gold Card holders but the total costs of their pharmaceuticals and total number of claims were similar. Possible explanations for this finding include: Gold Card holders made more visits to a GP – which we have discounted; GPs wrote more scripts per visit; and/or lower cost pharmaceuticals were prescribed. Our data did not allow us to analyse whether GPs wrote more scripts for the DVA Gold Card holders than women with no connection to DVA. However, in a survey of GP consultations (2000–2003), Britt et al [11] found that longer consultations were more likely for patients with a DVA Gold Card, and those who were older and female, but that the consultations resulted in fewer medications being prescribed and used. This practice by GPs could also reflect the influence of the DVA Prescriber Intervention and Feedback program (PFP) from 1993–2003, and/or its successor, the Veterans’ Medicines Advice and Therapeutics Education Service (Veterans’ MATES) programs. These programs assist GPs and veterans manage medicines in order to prevent medication-related problems such as adverse effects or drug interactions [12].

Another possible explanation for why these DVA Gold Card holders had more scripts but not higher total costs for pharmaceuticals is that they do not fill all prescription repeats, possibly indicating non-adherence. Hynd et al [13] reported significantly less dispensing of PBS prescriptions following the introduction of the co-payment price increase in 2005, the same year our data were collected. They found that pharmaceutical dispensing was 2 %-9 % less for people with ‘social security cards’ (including age concession cards) than for the general population. This change to the system, which would have also impacted on Gold Card holders, might partially explain our finding.

Few other studies have looked at similar issues. The most relevant is an Australian Institute of Health and Welfare [AIHW] report [14] which analyzed Australian medical and pharmaceutical usage by veterans and war widow(er)s who were Gold Card holders from 1997–2000 and showed that female Gold Card holders aged 70–84 years (mostly World War II widows) had an 11 % higher use of local medical officer/GP services than the rest of the Australian community, matched for age. Additionally, in 1999–2000 female DVA Gold Card holders aged 70–89 years had 4 %-8 % lower use of pharmaceuticals than similarly aged women in the Australian community. The authors raised the possibility of under-reporting of PBS claims if Gold Card holders did not use their Gold Cards or used other non-DVA concession cards when presenting scripts. We can discount under-reporting as our complete linkage to Medicare data enabled us to cross check the women’s DVA status and concession card status for all claims. The AIHW report found the cost per script to be 16 % lower for female Gold Card holders aged 70–79 years, and, like us, could not explain this apart from hypothesising that DVA Gold Card holders are prescribed less expensive drugs. However, comparisons with our study are not straightforward as the AIHW study did not separate widows and married women, restricted the MBS data to local medical officer and GP (out-of-hospital) services, and used pharmaceutical use data estimated from multiple sources because PBS(RPBS) data were incomplete at that time [14].

### Study limitations

The findings should be considered with respect to the limitations of the study. Only ALSWH participants who consented to data linkage were included. While the consent rates for data linkage for the two groups of women were similar (62 % of women with no connection to DVA versus 67 % of Gold Card holders), ALSWH participants who consent to linkage tend to be slightly better educated and better able to manage on their income than non-consenters [15,16]. This potentially creates a socioeconomic bias and underestimation of bulk billing rates [17]. Additionally, the numbers of Gold Card holders was relatively small, which may have prevented some important differences being identified and limited the number of covariates that could be used in the analyses. Our findings are strengthened by the fact that the ALSWH is a large, nationally representative random sample.

## Conclusions and Implications

In Australia, like the United States, the government shows its appreciation of the service and sacrifice of its war veterans and their spouses and dependents by providing significant ongoing support for their health care needs. This support is significant both in terms of cost and extent. In December 2011, there were an estimated 83,562 war widows of World War II veterans in Australia, and a further 77,400 World War II veterans receiving income support [18], many of whom will be outlived by their wives. Our results suggest older war widows are not using more medical services and pharmaceuticals than other older Australian women despite having financial incentives to do so. The main implication is that the current funding model is providing an equitable amount and type of support and access to health services to war widows compared to others. Given the strengths of the ALSWH, these findings can be viewed as being broadly representative of war widows in Australia. The extent to which such behaviour might be displayed by other demographic groups is unknown. Generalizations to other countries are more tenuous but the implications for the United States or the United Kingdom for example, are that spouses of older veterans do not necessarily exploit free services.

However, whether these results apply to policies for future funding models is uncertain. In Australia there is potentially an upcoming demographic of war widows of the estimated more than 47,100 men now aged 55 years and older who fought in the Vietnam War and who receive income support [18]. This cohort of women may well have a different sense of entitlement and attitudes to health service access and use than that displayed by the older generation of war widows.

## Competing interests

There are no competing interests to declare for any of the authors.

## Authors' contributions

LT contributed substantially to the conception and design and interpretation of the data and was principally responsible for drafting the manuscript. RH contributed substantially to the conception and design, data analysis and interpretation of the data, and reviewed drafts of the manuscript. ST contributed to the conception and design and interpretation of the data and reviewed drafts of the manuscript. CMc contributed to the conception and design and interpretation of the data and reviewed drafts of the manuscript. AD contributed substantially to the conception and design, interpretation of the data and to drafting the manuscript. All authors read and approved the final manuscript.

## Pre-publication history

The pre-publication history for this paper can be accessed here:

http://www.biomedcentral.com/1472-6963/12/179/prepub
